# Identification of an 88-microRNA signature in whole blood for diagnosis of hepatocellular carcinoma and other chronic liver diseases

**DOI:** 10.18632/aging.101253

**Published:** 2017-06-27

**Authors:** Xiao-Ran Long, Yao-Jun Zhang, Mei-Yin Zhang, Keng Chen, X.F Steven Zheng, Hui-Yun Wang

**Affiliations:** ^1^ State Key Laboratory of Oncology in South China, Sun Yat-Sen University Cancer Center, Guangzhou, Guangdong 510060, China; ^2^ Collaborative Innovation Center for Cancer Medicine, Sun Yat-Sen University Cancer Center, Guangzhou, Guangdong 510060, China; ^3^ Department of Hepatobiliary Oncology, Sun Yat-Sen University Cancer Center, Sun Yat-Sen University Cancer Center, Guangzhou, Guangdong 510060, China; ^4^ Department of Liver Disease, Guangzhou Eighth People’s Hospital, Guangzhou, Guangdong 510060, China; ^5^ Rutgers Cancer Institute of New Jersey and Department of Pharmacology, Robert Wood Johnson Medical School, Rutgers University, New Brunswick, NJ 08901, USA

**Keywords:** hepatocellular carcinoma, diagnosis, MicroRNA, microarray, alpha-fetoprotein

## Abstract

Hepatocellular carcinoma (HCC) is a common cancer with very poor survival due to lack of reliable biomarker for early diagnosis. In this study, we investigated microRNA (miRNA) profile of whole blood with a custom microarray containing probes for 1849 miRNA species in a total 213 successive subjects who were divided into a discovery set and a validation set. An 88-miRNA signature was established to diagnose health controls (HC), chronic hepatitis B (CHB), liver cirrhosis (LC) and HCC with 100% accuracy in the discovery set using Fisher discriminant analysis. This diagnostic signature was confirmed in the validation set with accuracy rates of 100%, 95.2%, 93.7% and 98.4% for HC, CHB, LC and HCC patients, respectively. Compared with AFP, the only available non-invasive and routinely used biomarker for diagnosis of HCC, the 88-miRNA signature has much higher accuracy (99.5% vs 76.5%), sensitivity (100% vs 63.8%), and specificity (99.2% vs 84.2%). More importantly, the signature detects small HCCs (<3cm) with 100% (17/17) accuracy while AFP has only 64.7% (11/17). In conclusion, we have identified a powerful and sensitive blood 88-miRNA signature for diagnosing early HCC and other chronic liver diseases (CHB and LC) with a high accuracy.

## INTRODUCTION

Hepatocellular carcinoma (HCC) is the third leading cause of death from cancer and the fifth most prevalent malignancy worldwide [1]. Although there are many advances in treatment, HCC patients still have very poor overall survival with 5-year survival rate below 12% [2]. The main reasons for such low survival rate of HCC are asymptomatic and diagnosed at advanced stages due to lack of accurate and non-invasive diagnostic tools for early detection of HCC [3], resulting in missing the best opportunity for curative surgery. In China, more than 401,000 new patients are diagnosed of HCC and more than 371,000 HCC patients die from this disease every year [4]. Furthermore, nearly 10% of the Chinese population is the carrier of hepatitis B virus (HBV) [5]. Approximate 10% of the patients with chronic hepatitis B (CHB) develop into liver cirrhosis (LC), the leading risk factor for HCC. In addition, some HCCs can directly arise from chronic hepatitis B virus (HBV) infection. Therefore, HCC diagnosis requires differentiation from CHB and LC. Currently, the main non-invasive methods for diagnosis of HCC include ultrasonography and AFP serology. Although serum Alpha-fetoprotein (AFP) has been used for decades as a diagnostic biomarker for HCC, it cannot be used as an independent diagnostic marker because of unsatisfactory sensitivity and specificity. For example, serum AFP level may be elevated in patients with CHB and LC also. Ultrasonography is a useful and non-invasive method for detection and surveillance of HCC, but it does not differentiate well between liver benign and malignant nodules, especially for the small ones (< 2cm) in patients with LC and/or HCC [6]. Therefore, there is an urgent need to identify novel, effective, sensitive, specific and non-invasive biomarkers for early diagnosis of HCC in order to improve the survival of HCC patients.

MicroRNAs (miRNAs) are a class of small (~21 nucleotide) noncoding RNAs that generally negatively regulate the expression of their target genes [7]. Dysregulation of miRNA expression is a common feature in human cancers including hepatocellular carcinoma (HCC) [8–10]. The circulating miRNAs in the plasma, serum or whole blood are considered to be ponderable, stable and noninvasive biomarkers for cancer diagnosis [11, 12]. Numerous tumor-derived miRNAs have been reported to be detected in the serum, plasma or blood of cancer patients, which are useful as diagnostic biomarkers for many cancers [13–16]. In 2010, Li L et al. reported the first study in which they first screen miRNAs in two pooled serum samples using Solexa sequencing, and then identified and validated two sets of serum miRNAs for diagnosis of HCC with high accuracies in larger serum sample size by quantitative RT-PCR [8]. Since then, numerous studies on circulating miRNAs (either panel of miRNAs or single miRNA) for diagnosis and prognosis of HCC have been reported [17–19]. However, other than Li’s report, so far there are only three diagnostic studies on the circulating miRNA profiling for diagnosis of HCC using high-throughput methods. First, in 2011, Zhou et al employed a microarray to screen 723 miRNAs in 137 plasma samples, established a 7-miRNA panel for diagnosing HCC in 407 plasma samples, and finally validated the panel of miRNAs in 390 samples with diagnostic accuracy of 89% [20]. In 2015, Wen et al applied TLDA Chips to screen 377 miRNAs in 9 plasma samples and identified an 8-miRNA panel as biomarkers for detection of HCC in discovery set (85 samples) and validation set (64 samples) with diagnostic accuracies of 82.3% and 78.0%, respectively [21]. In 2017, Zhu et al used deep sequencing to screen miRNAs in 100 serum samples and identified a 2-miRNA panel for diagnosing HCC with accuracies of 84.2% and 83.6% in training set and validation set, respectively [22]. However, these signatures remain unsatisfactory due to a low diagnostic accuracy of less than 90%. Furthermore, the reliability and feasibility of these signatures remain to be further validated in clinic. In addition, our experience shows that the quantity and quality of RNAs isolated with most commercial kits from serum or plasma is of poor yield and reproducibility, causing inconsistent results even with the same samples (data not published). The latter may explain why serum or plasma miRNAs are difficult to develop as biomarkers in clinical practice.

Recently, individual or set of miRNAs derived from whole blood sample has been reported as new biomarkers for early detection of pancreatic cancer [16, 23, 24], ovarian cancer [25], lung cancer [26–28], and gallbladder cancer [29]. miRNAs sourced from the whole blood including mononuclear cells can be used as diagnostic biomarkers based on the theory that circulating blood cells monitor the patients’ physiological and pathological state and respond by altering their transcriptome [30]. The advantages of whole blood miRNA samples are as follows: 1) high miRNA yield [31], 2) less error-prone than the serum or plasma samples, and 3) the whole blood samples contain both tumor-secreted miRNAs and other miRNAs that change following tumor progress, the inflammatory or immunoreactive stage, which yield more comprehensive information than the serum or plasma samples [24, 25]. Other than solid cancers, the whole blood miRNAs can be sourced from distant tissues such as inflammatory foci, neutrophils, monocytes, platelets, and mature red blood cells. Thus, they are more sensitive in inflammation-related cancers such as chronic pancreatitis related pancreatic cancer and HBV related HCC [24]. To our knowledge, there has not been any report on the diagnostic value of whole blood miRNAs in HCC patients to date.

Here, we present a multicenter study on the whole blood miRNA expression profile with a custom microarray in a total of 213 cases consisting of 43 healthy controls (HC), 45 chronic hepatitis B (CHB) patients, 45 liver cirrhosis (LC) patients and 80 HCC patients. In this study, we identified an 88-miRNA signature that accurately diagnose patients with HCC, CHB and LC in a discovery set (150 cases), which was confirmed in a validation set (63 cases).

## RESULTS

### Clinical characteristics of the patients

To profile miRNA expression in whole blood, we initially collected 150 blood samples as a discovery set for identification of diagnostic signature. After establishment of a diagnostic signature, we obtained another 63 blood samples as a validation set to verify the diagnostic signature. As shown in Table 1, Alanine aminotransferase (ALT), Aspartate transaminase (AST) and Globuline (GLOB) levels are significantly higher in patients with chronic liver diseases including CHB, LC and HCC compared with HCs, while albumin (ALB) is decreased in the patient groups, which indicates a typical liver damage in the patient groups. Among the patients, a significant percentage of HCC patients has higher levels of ALT and AST than those with CHB and LC indicating that these HCC patients already have liver damage. In addition, 97.5% of HCC patients have HBsAg positive, demonstrating that nearly all of HCC patients are infected with HBV virus. Finally, 63.7% of HCC patients have AFP positive, and 37.8% of CHB and 26.7% of LC patients also have AFP positive, suggesting that AFP is not a specific marker for HCC.

**Table 1:** Comparison of clinical characteristics of patients and controls

Clinical characteristics	HC (N = 43) n (%)	CHB (N = 45) n (%)	LC (N = 45) n (%)	HCC (N = 80) n (%)	*P* value
**Age (years)**	42.2 ± 8.7	43.3 ± 12.7	49.8 ± 12.5	49.0 ± 13.0	0.725^a^
≤ 40	18 (41.8)	19 (42.2)	10 (22.2)	50 (62.5)	0.109
> 40	25 (58.1)	26 (57.8)	35 (77.8)	30 (37.5)	
**Sex ratio**					
male	22 (51.2)	31 (68.8)	34 (75.6)	66 (82.5)	0.217^b^
female	21 (48.8)	14 (31.2)	11 (24.4)	14 (17.5)	
**ALT (U/L)**	18.5 ± 10.9	104.2 ± 200.7	57.5 ± 62.3	104.0 ± 64.1	<0.001^a^
≤ 40	42 (97.6)	24 (53.3)	22 (48.9)	12 (15.0)	<0.001^b^
> 40	1 (2.4)	21 (46.7)	23 (51.1)	68 (85.0)	
**AST (U/L)**	17.8 ± 5.6	42.7 ± 38.2	54.4 ± 45.8	77.3 ± 42.2	<0.001^a^
≤45	43 (100.0)	31 (68.9)	22 (48.9)	16 (20.0)	<0.001^b^
>45	0 (0.0)	14 (31.1)	23 (51.1)	64 (80.0)	
**ALB (g/L)**	39.5 ± 3.1	34.6 ± 3.8	32.7 ± 4.0	32.3 ± 3.4	<0.001^a^
≤ 35	3 (6.9)	31 (68.9)	34 (75.6)	61 (76.3)	<0.001^b^
> 35	40 (93.1)	14 (31.1)	11 (24.4)	19 (23.7)	
**GLOB (g/L)**	21.2 ± 6.9	31.0 ± 6.1	32.9 ± 5.3	32.5 ± 7.5	<0.001^a^
≤ 35	42 (97.7)	34 (75.6)	29 (64.4)	51 (63.8)	0.001^b^
> 35	1 (2.3)	11 (24.4)	16 (35.6)	29 (36.2)	
**HBsAg**					
Positive	N/A	45 (100.0)	43 (95.6)	78 (97.5)	0.003
Negative	N/A	0 (0.0)	2 (4.4)	2 (2.5)	
**HBV DNA (IU/ml)**	N/A	1.6×10^5^ ± 5.7×10^5^	2.9×10^6^ ± 8.3×10^6^	6.4×10^5^ ± 2.1×10^6^	0.009^a^
< 10^3^	N/A	1 (2.2)	5 (11.1)	6 (7.5)	0.309
> 10^3^	N/A	44 (97.8)	39 (88.9)	74 (92.5)	
**AFP (μg/L)**	21.0 ± 7.4	39.1 ± 58.5	49.9 ± 90.5	2106.4 ± 3401.4	<0.001^a^
≤ 25	43 (100.0)	28 (62.2)	33 (73.3)	29 (36.3)	<0.001^b^
> 25	0 (0.0)	10 (37.8)	11 (26.7)	51 (63.7)	

^a^*P* value of one-way ANOVA; ^b^ Chi-square test; N/A, data is not available

Abbreviation: HC, health control; CHB, chronic hepatitis B; LC, liver cirrhosis; HCC, hepatocellular carcinoma;

ALT, Alanine aminotransferase; AST, Aspartate transaminase; GLOB, Globuline; ALB, albumin; AFP, Alpha-fetoprotein.

### MicroRNA expression profiles of whole blood from HCs and patients with CHB, LC and HCC in the discovery set and verification of microarray data by qRT-PCR

**Figure 1:**
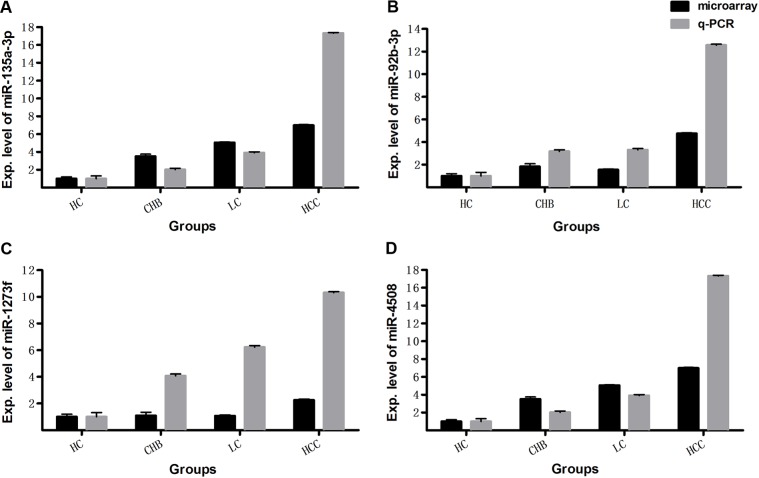
The expression levels of miRNAs detected by microarray were validated with qRT-PCR The relative expression levels of miR-135a-3p (**A**), miR-92b-3p (**B**), miR-1273f (**C**), miR-4508 (**D**) were examined by qRT-PCR in another 40 whole blood samples consisting of 10 HC, 10 CHB, 10 LC and 10 HCC subjects, and compared with microarray data in the same 40 samples. The qRT-PCR reaction of each sample was performed in triplicate and the mean values were calculated. Relative expression levels are presented with the mean value of qRT-PCR or microarray data in 10 samples of each group.

In this study, we investigated miRNA expression profiles from whole blood in a total of 213 cases of HC, CHB, LC and HCC subjects. With SAM program and student t test, we found that there are 275 differentially expressed miRNAs with >1.5-fold change between HCs and patients with CHB, LC and HCC (q-value (%) = 0), in the discovery set 231 of which are up-regulated, and 44 down-regulated in the patients. To validate the microarray results, miR-4508, miR-135a-3p, miR-1273f and miR-92b-3p were examined by qRT-PCR in 40 plasma samples consisting of 10 HC, 10 CHB, 10 LC and 10 HCC subjects randomly selected from the discovery set. Quantitative RT-PCR results showed that the four miRNAs are upregulated in patients with CHB, LC and HCC compared with HCs (Fig. 1), which is consistent with the results obtained by microarray analysis. These results demonstrate the reliability and reproducibility of the microarray data.

### Identification of an 88-miRNA diagnostic signature in the discovery set

**Figure 2:**
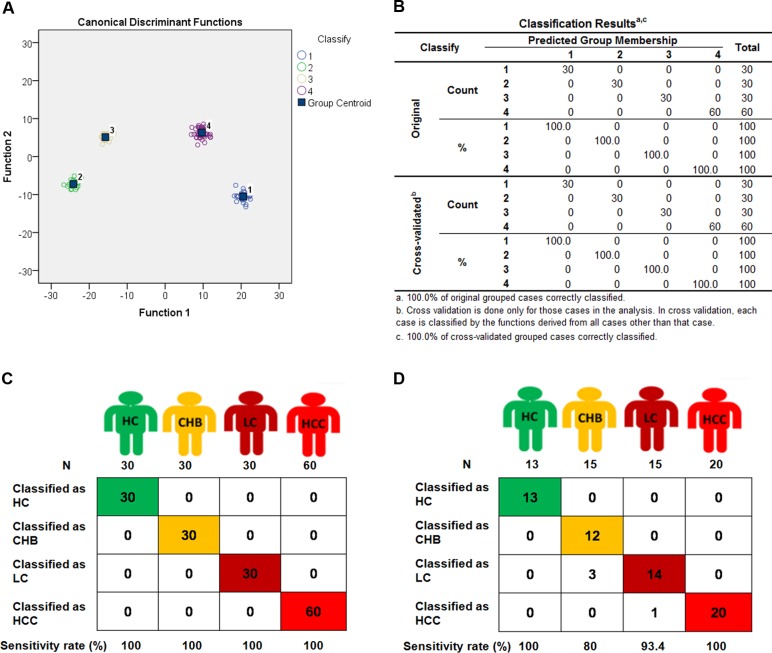
The 4 different groups were classified by 88-miRNA signature in discovery and validation sets Fisher discriminant analysis (Stepwise discriminant method) of Health control (HC), chronic hepatitis B (CHB), liver cirrhosis (LC) and hepatocellular carcinoma (HCC) subjects was performed to establish a 88-miRNA signature in the discovery set. With the 88-miRNA signature, HC, CHB, LC and HCC groups were classified and presented with classification plot (**A**), classification table (**B**), and classification sketch (**C**) in the discovery set. (**D**) The HC, CHB, LC and HCC groups were classified by the signature in validation set and presented with classification sketch.

Upon profiling miRNA expression in whole blood samples, we employed the 275 differentially expressed miRNAs to identify signatures with diagnostic value for HC (30 subjects), CHB (30 subjects), LC (30 subjects), and HCC (60 subjects) in the Discovery set. Fisher discriminant analysis (Stepwise discriminant method) was used to find the best combination of miRNAs that can distinguish the four groups of HC, CHB, LC, and HCC. An 88-miRNA diagnostic signature (Supporting Table 1) was identified in the discovery set. For diagnosing the four different subjects, four Fisher’s discriminant formulas were constructed based on the 88 miRNA expressions: score_(i)_ = constant_(i)_ + ∑ coefficients_(i)_ * miRNA expression values. In the formula, (i) represents HC, CHB, LC or HCC. In the four formulas, the same miRNA expression value of each subject multiplies 4 different coefficients for HC, CHB, LC and HCC. In general, HCs have the lowest score of the 88 miRNAs and HCCs have the highest score among the four groups. With the four formulas, four diagnostic scores were calculated for each subject. If the highest score was presented in the formula for HC, the subject was predicted as HC; if the highest score is in the formula for HCC, the subject was predicted as HCC, and all subjects could be predicted in the same manner (Supporting Table 2). Interestingly, the 88-miRNA signature correctly diagnosed all 150 subjects including HCs, CHBs, LCs and HCCs with 100% accuracy (Fig. 2A - 2C).

### Verification of the 88-miRNA diagnostic signature in the validation set

To further verify this signature, we collected another 63 blood samples as a validation set to test the diagnostic reproducibility. These samples were detected with the same miRNA microarray. The same four formulas of the 88-miRNA signature obtained from the discovery set were used to calculate the diagnostic score for each subject in the validation set (Supporting Table 3). As expected, the diagnostic accuracy rates are 100% (Sensitivity [Se]: 100%, Specificity [Sp]: 100%), 95.2% (Se: 80.0%, Sp: 100%), 93.7% (Se: 93.3%, Sp: 93.8%) and 98.4% (Se: 100%, Sp: 97.7%) for HC, CHB, LC and HCC, respectively (Fig 2D), which are very similar to those results obtained in the discovery set, especially for HCs and HCCs with 100% sensitivity in both sets. These results indicate that the 88-miRNA signature is a powerful and reproducible diagnostic biomarker for CHB, LC and HCC patients.

### The diagnostic value of the 88-miRNA signature is much better than AFP for HCC

**Figure 3:**
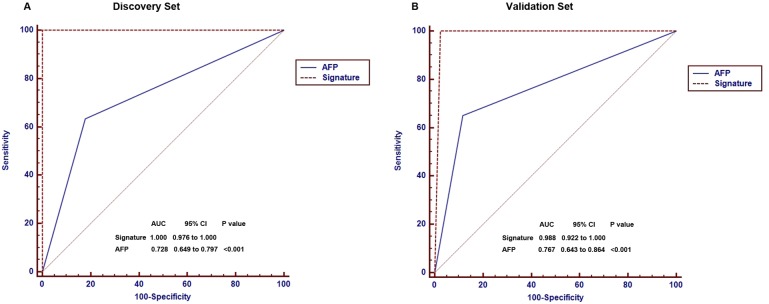
Comparison of diagnostic accuracies of the blood 88-miRNA signature and serum AFP for HCCs and non-HCC subjects in discovery and validation sets (**A**) The diagnostic accuracies of the blood 88-miRNA signature and serum AFP was compared by Receiver operating characteristic (ROC) analysis in the discovery set. (**B**) The diagnostic accuracies of the blood 88-miRNA signature and serum AFP by ROC analysis in the validation set.

In clinical practice, AFP is the only available biomarker routinely used as a non-invasive method for the diagnosis of HCC as a non-invasive method. However, the diagnostic sensitivity of AFP for HCC is only 60-70% [32, 33]. To validate whether the 88-miRNA signature is superior to AFP, we compared both markers by receiver operating characteristic (ROC) analysis. The ROC curves demonstrated that 88-miRNA signature has a much higher diagnostic accuracy for HCC (area under the curve [AUC]: 1.000) than AFP (AUC: 0.728, P<0.001) in discovery set (Fig 3A). This result was further verified in the validation set (signature vs AFP, AUC: 0.988 vs 0.767, P<0.001, Fig 3B).

To directly demonstrate that the 88-miRNA signature is superior to AFP for diagnosis of HCC, we compared the two markers in all of the 213 subjects. The result indicates that the 88-miRNA has 99.5% (212/213) accuracy, 100% (80/80) sensitivity and 99.3% (132/133) specificity for diagnosis of HCC, while AFP only has 78.9% (163/213) accuracy, 63.8% (51/80) sensitivity and 86.8% (112/133) specificity (Table 2, Supporting Table 4). These results demonstrate that the 88-miRNA signature is a more powerful, sensitive and reproducible biomarker for HCC than AFP.

**Table 2:** Comparison of HCC diagnostic efficiency of blood 88-microRNA signature and serum AFP on all of 213 subjects

	88-microRNA signature	AFP
**Sensitivity % (n/n)**	100.0 (80/80)	63.8 (51/80)
**Specificity % (n/n)**	99.2 (132/133)	84.2 (112/132)
**Accuracy % (n/n))**	99.5 (212/213)	76.5 (163/213)
**Positive predictive value % (n/n)**	98.8 (80/81)	70.8 (51/72)
**Negative predictive value %(n/n)**	100 (132/132)	79.4 (112/141)

More importantly, the 88-miRNA signature correctly diagnosed all of the 17 HCC patients whose tumors are less than 3 cm (median 2.3 cm, ranging from 1.2 to 2.9 cm). In contrast, AFP only correctly determined 64.7 percent (11/17) of the patients with small tumor (Median 2.7, ranging from 1.5 to 2.9; Table 3). These results indicate that the 88-miRNA signature can benefit early diagnosis of HCC.

**Table 3:** Comparison of serum 88-microRNA signature and AFP for diagnosis of small HCC (<3.0 cm)

		HCC Size (cm)	Signature score				Signature diagnosis	AFP	AFP diagnosis
No	ID		Predict value for HC	Predict value for CHB	Predict value for LC	Predict value for HCC		Conc. (ng/mL)	
1	10	1.7	13410	10316	13160	13867	HCC	18.0	No
2	11	1.6	10013	9145	11110	11001	HCC	128.4	HCC
3	16	2.3	400	1686	213	2726	HCC	7.0	No
4	45	2.9	15218	12848	15514	15547	HCC	9307.3	HCC
5	24	2.8	13948	11916	14356	14416	HCC	3294.3	HCC
6	29	1.7	7471	7461	10112	8432	HCC	229.1	HCC
7	31	1.3	5789	4763	5717	6328	HCC	23.8	No
8	36	2.4	7581	6938	7086	8345	HCC	552.2	HCC
9	37	2.9	3207	2819	4524	4726	HCC	1631.7	HCC
10	39	2.9	2854	2364	4123	4346	HCC	6782.6	HCC
11	44	1.5	4264	4920	4055	5037	HCC	41.4	HCC
12	54	2.3	5104	5354	4732	5848	HCC	23.2	No
13	55	2.7	10239	8631	10287	10487	HCC	528.9	HCC
14	73	1.8	8687	7174	8593	8883	HCC	639.5	HCC
15	77	1.2	8816	7287	8779	9035	HCC	9.6	No
16	78	2.7	8442	6805	8130	8775	HCC	337.5	HCC
17	84	2.2	7215	5451	6244	9039	HCC	23.5	No

Abbreviation: Conc., concentration; HC, health control; CHB, chronic hepatitis B; LC, liver cirrhosis; HCC, hepatocellular carcinoma; AFP, Alpha-fetoprotein.

## DISCUSSION

With the advance in high-throughput detection techniques for miRNAs, more and more circulating miRNAs have been found to correlate with cancer diagnosis, progression, prognosis and treatment response, indicating that these miRNAs have great potential for improving diagnosis, prognosis and therapy in cancer patients [34]. In the normal population, the composition of circulating miRNAs most closely correlates with that of liver miRNAs [35], suggesting that under normal conditions liver is the main source for circulating miRNA. Therefore, when lesions (including cancers and HBV infection) occur in the liver, the composition of circulating miRNAs change accordingly, allowing liver diseases to be detected by profiling blood miRNAs.

In this study, we performed a multicenter study on blood miRNA profiles of chronic liver diseases with a custom microarray including 1849 miRNA species, which allowed us identify a diagnostic signature for patients with CHB, LC and HCC. In the miRNA profiling, there are 231 upregulated miRNAs and 44 downregulated ones in patients compared with health controls. The reason for the predominant upregulated miRNAs may be that during the carcinogenesis, more miRNAs are expressed to inhibit the expression of tumor suppressor genes. In contrast, few oncogenes are activated in this manner. It is known that more miRNAs have increased in human cancer [36, 37].

In the discovery phase, an 88-miRNA diagnostic signature was established in a total of 150 available cases, which correctly diagnosed all the 150 cases including HC, CHB, LC and HCC with 100% accuracy. Then we collected another 63 cases consisting of 13 HCs, 15 CHBs, 15 LCs and 20 HCCs from two different medical centers as a validation set. In the validation, the 88-miRNA signature also achieved high diagnostic accuracy: 100% for HC, 95.2% for CHB, 93.7% for LC, and 98.4% for HCC. These results indicate that we for the first time establish a blood 88-miRNA diagnostic signature with high accuracy and reproducibility for chronic liver diseases associated with HBV infection.

Early diagnosis of HCC is critical for enhancing patient survival. Serum AFP was first introduced as diagnostic marker for primary liver cancer in 1964. Since then it has been used for screening and diagnosing HCC worldwide for more than 50 years [38, 39]. However, it has been recognized that single AFP marker has an unsatisfactory sensitivity for detection of HCC because nearly 33% of HCC patients do not have elevated serum AFP level [40]. The specificity of serum AFP also suffers due to the fact that many patients with benign diseases also have an elevated AFP level. For example, although Zhang et al reported that AFP plus ultrasound surveillance every 6 months in a population with HBV infection significantly reduced HCC mortality by 37% compared with a non-screened population with HBV infection [39], another similar study showed that HBV carriers with periodic AFP screening had no survival benefit compared to those without screening [41]. Therefore, the American Association for the Study of Liver (AASLD) guidelines do not recommend serum AFP surveillance for HCC unless ultrasound is unavailable [6, 42]. Therefore, considerable efforts have been made on finding better serum surrogate markers for HCC than AFP over the last several decades. However, no new surrogate marker for diagnosis of HCC is superior to serum AFP in clinical practice. In this study, we present a blood 88-miRNA signature with 100% and 98.4% diagnostic accuracies for HCC patients in discovery set and validation set, respectively. This is in contrast to 72.8% and 76.7% accuracies for serum AFP in discovery set and validation set. Thus, the blood 88-miRNA signature is a powerful and reproductive surrogate for patients with HCC. Furthermore, the blood 88-miRNA signature can correctly detect 100% (17/17) HCC patients with tumor size less than 3 cm (median: 2.3 cm, ranging: 1.2 - 2.9 cm). In contrast, AFP only diagnose 61.5% (8/13) HCC patients (median: 2.7 cm, ranging: 1.5 -2.9 cm). These results indicate that our blood 88-miRNA signature can lead to early HCC diagnosis of HCC and hence better patient survival. Further studies on small HCC (< 1 cm) detection with the signature are necessary before this blood 88-miRNA signature can be applied in routinely clinical practice. The test also needs to be further verified in larger more HCC patient population and more medical settings.

In early detection of HCC, distinguishing HCC from LC is a big challenge because the nodule configuration of cirrhosis is very similar to that of HCC. Moreover, both HCC and LC patients have elevated AFP level. Worldwide, ultrasound as the main method for HCC surveillance is recommended every 6 months for patients with cirrhosis to increase the early detection rate and survival rate of HCC patients [42]. However, one -fourth of early HCC patients fail to be detected by ultrasonography in early stage HCC patients with cirrhosis [43]. Furthermore, ultrasonography does not distinguish well benign nodules from malignant ones in patients with cirrhosis. In contrast, our blood 88-miRNA signature not only diagnoses HCC with nearly 98.4 - 100% accuracy, but also detects HCC as small as 1.2 cm in diameter. More importantly, this signature can also diagnose liver cirrhosis with 93.7% accuracy. These results suggest that the blood 88-miRNA signature is a potentially powerful biomarker for early screening and diagnosis of HCC.

In summary, we for the first time analyzed the miRNA expression profiles of whole bloods from subjects of HC, CHB, LC and HCC, and established and validated an blood 88-miRNA signature that diagnose CHB, LC and HCC with high accuracies in discovery and validation sets, respectively, which may be a powerful non-invasive biomarker for early diagnosis of HCC patients.

## MATERIALS AND METHODS

### Patients and tissue samples

A total of 213 cases (containing 43 healthy participants, 45 chronic hepatitis B patients, 45 cirrhosis patients and 80 HBV-related HCC patients) were recruited for this study. Of these subjects, 80 HBV-related HCC patients collected from the Sun Yat-Sen University Cancer Center, during January 2015 to December 2016, were diagnosed as HCC by pathological examination and did not have any treatments before surgery. The 45 CHB and 45 LC samples were obtained from the Guangzhou Eighth People’s Hospital in Guangzhou, where patients with CHB were diagnosed when patients had serum HBsAg positive for more than 6 months, and patients with cirrhosis were diagnosed by liver biopsy. The 43 healthy participants’ samples were collected from the Health Examination Center of the Sun Yat-Sen University Cancer Center. All of these samples were sequentially collected and the first 150 samples assigned to a discovery set (containing 30 healthy participants, 30 chronic hepatitis B patients, 30 cirrhosis patients and 60 HBV-related HCC patients) and the second batch of 63 samples into a validation set (containing 13 healthy participants, 15 chronic hepatitis B patients, 15 cirrhosis patients and 20 HBV-related HCC patients). This study was reviewed and approved by the Ethical Committees of Sun Yat-Sen University Cancer Center and Guangzhou Eighth People’s Hospital. The written informed consent was obtained from each patient.

### RNA extraction

RNA was extracted from 2-3ml of whole blood sample obtained from each patient using the Blood RNA Preservative Tubes and RNA Purification Kit (Norgen Biotek, Thorold, Ontario, Canada) according to the manufacturer’s protocol. Briefly, whole blood was mixed well with 1.5 ml of RNA Extraction Buffer A and 1.5 ml of RNA Extraction Buffer B; After incubated in -20°C for 10 minutes, the mixture was centrifuged at 4°C at 4000 RPM for 30 minutes; After the supernatant was discarded, 570 μL of Resuspension Solution B and 330 μL of 100% Ethanol were added and mixed well; The mixture was added into the Mini Spin column, and then the column was centrifuged at 4°C at 3500 RPM for 1 minute; After the column was washed with Wash Solution A for three times, 100 μL of Elution Solution A was added into the column and centrifuged at 4°C at 1000 RPM for 2 minutes, followed by 2 minutes at 4500 RPM to elute the RNA sample. Finally, RNA in 100 μL was concentrated to 20 μL, and RNA concentration was measured in an ND-1000 spectrophotometer (NanoDrop Technologies).

### Microarray detection

All 1921 human mature miRNAs in the miRBase database (Release 18.0) were used for designing probes for constructing the in-house miRNA microarray and a total of 1849 probes have been successfully designed according to the principle proposed by Wang [44]. The microarray was fabricated in house and hybridized as described by us previously [45, 46]. Briefly, each probe was mixed with printing buffer to a final concentration of 40 μmol/L and printed in duplicate on the cleaned glass slides (75 × 25 mm). The total RNA (1.0 -1.5 μg) was labeled with 100 nmol/L of pCp-Cy5 (Jena Bioscience, Germany) and 15 units of T4 RNA ligase (USB) in a total reaction volume of 20 μL at 16°C overnight. Then the mixture of labeled RNA sample and 1x hybridization solution was hybridized onto the microarray for 12 -18 h at 45°C. After hybridization, the slides were washed in 1×SSC/1% SDS for 10 min at 45°C, followed by sequential washing in 2 cycles of 0.5 ×SSC/0.1% SDS, 2 cycles of 0.2×SSC and 1 cycle of purified water for 1 min at room temperature, respectively, and then dried in a special small centrifuge and scanned using the LuxScan-10K (CapitalBio, China).

### Gene expression data extraction

The microarray scanning images were digitized with GenPix Pro 6.0 program, and the raw signal data were extracted, subtracted background and normalized (Quantile normalization) using GPR analysis software (edited in-house). Then we computed the average intensity of the repetitive probes and transformed them into log2 value. The microarray data have been deposited in Gene Expression Omnibus of the National Center for Biotechnology Information (GSE53882).

### Quantitative RT-PCR

For qRT-PCR, total RNA (10 ng) was reversely transcribed with TaqMan Assays (Thermo Fisher) including miRNA-specific reverse transcription-primers and MultiScribe Reverse Transcriptase. Quantitative PCR reactions were performed with Universal PCR Master Mix II (TaqMan) on a PRISM 7900HT system (Applied Biosystems) with U6 RNA as the internal control. Each sample was analyzed in triplicate wells, and reactions without cDNA also were included as negative control. The conditions of thermal cycling were as follows: 95°C at 10 min for a hot start, then 40 cycles at 95°C for 15 s, 60°C for 60 s. U6 RNA was used as loading control. The PCR data were first normalized by U6 expression and then by the median expression value of a given microRNA in the corresponding subjects. The relative quantification (RQ) of microRNA expression was presented as 2^−ΔΔCt^.

### Statistical analysis

We used student’s t test and significance analysis of microarray (SAM) to identify the differentially expressed miRNAs (fold changes >1.5, P<0.001 and FDR-q <0.05) between healthy subjects and patients’ subjects. The differentially expressed miRNAs were used to establish diagnostic miRNA signatures that can distinguish the four groups of HC, CHB, LC, and HCC using Fisher Discriminant Analysis [47] in SPSS Version 20.0 software, and receiver operating characteristics (ROC) analyses were performed to compare the diagnostic accuracies of 88-miRNA signature and AFP in Stata software.

## SUPPLEMENTARY MATERIAL


