# Supplementary material for: Identification of an 88-microRNA signature in whole blood for diagnosis of hepatocellular carcinoma and other chronic liver diseases
Source: Aging (Albany NY). 2017 Jun 27;9(6):1565–76. doi: 10.18632/aging.101253 (PMC5509456; doi:10.18632/aging.101253)
Supplement: Supplementary file 1 [file aging-09-1565-s001.pdf]

Supporting Table 1. The expression levels of 88 miRNAs of HC, CHB, LC, and HCC groups in the discovery set.

miRNA	Average value in HC	Average value in CHB	Average value in LC	Average value in HCC	Change fold (Patients/HC)
hsa-let-7a-2-3p	405	4610	2588	662	5.33
hsa-let-7d-5p	2498	21293	15304	8307	5.50
hsa-let-7g-3p	3443	12270	6735	7283	2.50
hsa-miR-103a-3p	8509	3498	3856	3109	0.40
hsa-miR-1247-5p	414	16697	12440	3681	12.03
hsa-miR-1248	405	4879	3602	838	6.17
hsa-miR-126-3p	1306	13331	11640	2144	5.58
hsa-miR-130a-3p	11153	3562	1444	3957	0.27
hsa-miR-132-5p	1780	13949	9531	3249	4.45
hsa-miR-150-5p	1459	45079	37385	10436	17.98
hsa-miR-1537	2417	14258	6090	8928	4.29
hsa-miR-154-3p	2865	31662	22702	5106	6.03
hsa-miR-18b-5p	2035	11121	4390	4276	3.09
hsa-miR-194-3p	1255	8254	3054	1499	3.22
hsa-miR-199a-5p	8029	20463	13660	8748	2.28
hsa-miR-19b-1-5p	8566	3145	1822	3893	0.34
hsa-miR-221-3p	1201	12836	13183	1510	7.19
hsa-miR-23a-5p	625	10196	7067	1163	8.44
hsa-miR-25-5p	592	8282	3755	803	1.63
hsa-miR-2681-5p	481	15051	12339	2372	6.08
hsa-miR-30b-3p	1317	15181	13914	2884	16.95
hsa-miR-30c-1-3p	737	27879	28071	6565	6.69
hsa-miR-30d-3p	1985	11928	12472	4393	11.68
hsa-miR-3145-5p	756	1547	13551	27001	4.11
hsa-miR-3146	703	10723	9364	1159	2.26
hsa-miR-3153	513	14634	14254	3146	8.01
hsa-miR-3162-5p	692	11473	13051	3257	17.54
hsa-miR-3164	1380	9920	10684	2111	11.08
hsa-miR-3184-5p	529	4644	3127	1463	4.47
hsa-miR-3196	412	515	2294	4346	5.57
hsa-miR-34a-3p	928	7841	6115	1635	2.31
hsa-miR-3672	9561	1575	1360	4180	0.50
hsa-miR-3675-3p	4327	4987	4155	14690	4.71
hsa-miR-371b-5p	28252	970	636	15277	0.31
hsa-miR-374c-3p	488	16467	16424	3765	4.30
hsa-miR-378a-5p	11277	33246	36931	13305	2.27
hsa-miR-3908	1094	15529	14541	3373	11.09
hsa-miR-3935	537	14102	9829	3627	2.17
hsa-miR-4284	441	7075	6539	725	8.57
hsa-miR-431-3p	926	26394	21277	10260	15.13

**hsa-miR-4322**	504	11689	6945	1054	8.76
**hsa-miR-4329**	3599	37035	39209	11812	6.47
**hsa-miR-4418**	612	10967	9371	1956	10.69
**hsa-miR-4444**	673	10185	8032	1895	11.47
**hsa-miR-4446-3p**	1646	13211	11880	1929	9.99
**hsa-miR-4461**	524	9177	6991	856	8.21
**hsa-miR-4472**	492	12258	7935	1497	6.54
**hsa-miR-4474-3p**	501	16540	17177	2575	8.92
**hsa-miR-4476**	472	7507	5536	1044	12.12
**hsa-miR-4478**	779	9952	8633	5058	19.50
**hsa-miR-4482-5p**	484	939	1150	3148	8.25
**hsa-miR-4484**	616	5354	6011	1445	10.65
**hsa-miR-4495**	506	10578	11778	1843	2.24
**hsa-miR-449a**	524	10721	10284	1371	14.23
**hsa-miR-4502**	2749	15664	15244	18080	12.98
**hsa-miR-4507**	691	12581	11140	2335	11.46
**hsa-miR-4508**	535	15235	10249	5958	6.06
**hsa-miR-450a-5p**	515	13725	10434	5608	10.51
**hsa-miR-450b-5p**	465	10215	8594	1541	18.76
**hsa-miR-4515**	1217	14161	13311	3235	18.03
**hsa-miR-4516**	1285	24272	24816	6728	12.06
**hsa-miR-4522**	2999	16253	15327	3679	7.13
**hsa-miR-4640-3p**	2943	859	715	7431	7.18
**hsa-miR-4646-3p**	1020	9174	10260	1556	2.49
**hsa-miR-4652-3p**	31329	1984	1407	10945	0.25
**hsa-miR-4677-5p**	534	6339	3400	975	6.03
**hsa-miR-4706**	1296	16999	14658	1500	9.19
**hsa-miR-4715-5p**	583	10523	8492	816	5.44
**hsa-miR-4732-5p**	459	7080	5134	810	8.89
**hsa-miR-4739**	439	3576	4135	1773	8.95
**hsa-miR-4750**	570	11876	9776	1247	7.54
**hsa-miR-4793-5p**	931	12926	10451	2639	6.34
**hsa-miR-485-5p**	2126	13614	14122	2208	11.43
**hsa-miR-493-5p**	1865	7491	3836	6130	7.92
**hsa-miR-5092**	620	19903	19179	4477	3.81
**hsa-miR-5096**	3611	3844	11017	32195	3.20
**hsa-miR-526b-5p**	526	5882	4993	862	5.50
**hsa-miR-541-5p**	537	6991	6083	843	9.41
**hsa-miR-618**	645	30247	22644	4228	7.23
**hsa-miR-644b-3p**	513	40087	39141	8967	6.37
**hsa-miR-662**	812	12058	10642	2518	8.51
**hsa-miR-767-3p**	967	2975	2761	5681	7.33
**hsa-miR-767-5p**	5182	23261	23596	6076	4.64
**hsa-miR-769-5p**	5511	41482	40317	14841	8.16
**hsa-miR-876-3p**	28942	43483	39932	49376	8.64
**hsa-miR-876-5p**	10690	5379	4989	5926	0.41
**hsa-miR-888-3p**	3676	3140	1383	5827	2.91
**hsa-miR-891b**	961	563	7496	7773	5.37

Supporting Table 2. The predicted result of 4 groups with 4 formulas of the 88-microRNA signature in the training set.

Sample	Group	Predict value for HC	Predict value for CHB	Predict value for LC	Predict value for HCC	Sensitivity (%)
1	HC	**4552**	4178	4265	4284	
2	HC	**4504**	4384	4489	4454	
3	HC	**8463**	6548	7801	8143	
4	HC	**9632**	7446	8975	9418	
5	HC	**15401**	12944	14413	15242	
6	HC	**15665**	12893	15566	15032	
7	HC	**19610**	15261	18812	18791	
8	HC	**15262**	12667	15218	14618	
9	HC	**16200**	13130	15917	15578	
10	HC	**17159**	13400	16289	16308	
11	HC	**16710**	13182	16015	16100	
12	HC	**20816**	16000	19704	20421	
13	HC	**16563**	13264	16109	15978	
14	HC	**17101**	13753	16696	16458	
15	HC	**6141**	4746	5464	5803	
16	HC	**8651**	6566	8010	8603	**100**
17	HC	**11657**	9837	10276	10457	
18	HC	**9224**	7219	8768	9187	
19	HC	**9043**	6740	8198	8818	
20	HC	**8516**	6368	7752	8336	
21	HC	**9360**	7399	8877	9290	
22	HC	**9070**	6730	8305	8940	
23	HC	**10033**	7518	9285	9973	
24	HC	**6583**	5702	6537	6314	
25	HC	**8398**	6698	8111	8147	
26	HC	**8290**	7228	7697	7463	
27	HC	**7132**	5165	6150	6827	
28	HC	**13480**	11213	13006	13374	
29	HC	**8834**	6867	8483	8705	
30	HC	**10678**	8326	10122	10613	
31	CHB	8030	**10835**	9766	10648	
32	CHB	10620	**10656**	9645	7998	
33	CHB	8032	**10841**	9768	10648	
34	CHB	3218	**3534**	3074	3498	
35	CHB	7273	**7395**	7206	6591	**100**
36	CHB	3650	**3720**	3228	3715	
37	CHB	3645	**3723**	3226	3716	
38	CHB	3305	**3593**	3156	3592	

39	CHB	5315	**7438**	5931	6374
40	CHB	2955	**3785**	2485	2844
41	CHB	4523	**4565**	3962	2986
42	CHB	6551	**7600**	6839	6490
43	CHB	6609	**9424**	7196	7883
44	CHB	5095	**5694**	5006	4309
45	CHB	4996	**5182**	4901	4293
46	CHB	7942	**8617**	7814	7439
47	CHB	2880	**3766**	3213	3014
48	CHB	3775	**5248**	3665	3795
49	CHB	1489	**1827**	692	653
50	CHB	3192	**4205**	2738	3390
51	CHB	13992	**14170**	14126	11647
52	CHB	14149	**14313**	14019	11523
53	CHB	19042	**19168**	18139	14635
54	CHB	8124	**10642**	9015	8189
55	CHB	5688	**7949**	7311	5784
56	CHB	5806	**8147**	7474	5916
57	CHB	5889	**6766**	6141	5218
58	CHB	-837	**843**	-732	-1497
59	CHB	-1603	**-337**	-1957	-2687
60	CHB	2599	**3261**	2861	3093
61	LC	3403	3593	**5839**	4162
62	LC	10686	9267	**12490**	11012
63	LC	5061	4702	**6919**	6564
64	LC	4941	4941	**5928**	4727
65	LC	310	1564	**2233**	402
66	LC	6524	6218	**7446**	6378
67	LC	5745	5345	**6544**	5374
68	LC	4132	4017	**4407**	3038
69	LC	4175	4072	**5883**	5050
70	LC	5008	5322	**5516**	4357
71	LC	4672	4954	**5360**	4108
72	LC	4421	4257	**6142**	5332
73	LC	3648	3989	**5148**	3377
74	LC	3788	3873	**5010**	3684
75	LC	3307	3824	**3900**	1988
76	LC	3665	3677	**4493**	2213
77	LC	5409	5433	**5445**	4569
78	LC	6663	5681	**9574**	8485
79	LC	5203	5085	**6413**	4692
80	LC	-3694	-4278	**-2480**	-5655
81	LC	-1628	-1021	**591**	-2288
82	LC	15924	12922	**16188**	16177

100

83	LC	14496	12149	**14745**	14630
84	LC	13017	11159	**13235**	13154
85	LC	12652	10431	**12780**	12373
86	LC	6354	7772	**8623**	6385
87	LC	9104	9612	**11448**	9356
88	LC	6255	8182	**9009**	6428
89	LC	8271	7746	**8767**	8268
90	LC	4638	4647	**5454**	4812
91	HCC	13410	10316	13160	**13867**
92	HCC	10013	9145	11110	**11001**
93	HCC	15483	12301	15487	**16531**
94	HCC	14641	10974	13978	**15339**
95	HCC	2706	2199	2831	**2968**
96	HCC	6307	5400	5988	**6863**
97	HCC	400	1686	212	**2726**
98	HCC	4614	3272	2818	**6317**
99	HCC	11349	9994	11160	**12202**
100	HCC	7670	6280	6390	**8935**
101	HCC	7343	5606	6421	**9136**
102	HCC	7370	6309	7256	**8953**
103	HCC	13666	11880	14194	**14257**
104	HCC	13948	11916	14356	**14416**
105	HCC	15522	13139	15882	**16167**
106	HCC	15822	13245	16093	**16453**
107	HCC	24717	18090	22489	**25030**
108	HCC	20723	16102	19479	**20969**
109	HCC	7471	7461	10112	**8432**
110	HCC	6215	7521	6031	**8733**
111	HCC	5789	4763	5717	**6328**
112	HCC	7495	7523	7411	**8746**
113	HCC	6778	5906	6927	**7218**
114	HCC	4645	5094	4124	**5641**
115	HCC	7581	6938	7086	**8345**
116	HCC	3207	2819	4524	**4726**
117	HCC	2854	2364	4123	**4346**
118	HCC	6013	6407	5765	**7273**
119	HCC	5992	5676	5771	**6331**
120	HCC	6398	5846	6258	**6689**
121	HCC	4723	4856	4453	**5015**
122	HCC	4264	4920	4055	**5037**
123	HCC	10174	9750	10384	**11289**
124	HCC	5104	5353	4732	**5848**
125	HCC	10239	8631	10287	**10487**
126	HCC	10079	10325	9886	**12011**

100

127	HCC	11982	10831	12217	**13024**
128	HCC	6397	6641	6407	**7133**
129	HCC	8151	8415	8210	**9563**
130	HCC	9516	8229	9740	**9766**
131	HCC	8488	7150	8559	**8714**
132	HCC	8264	6829	8237	**8698**
133	HCC	8324	6895	8139	**8518**
134	HCC	11699	9787	11747	**12082**
135	HCC	8348	7012	8407	**8620**
136	HCC	7299	6133	7399	**7768**
137	HCC	8693	7287	8682	**8936**
138	HCC	5657	5325	6015	**6270**
139	HCC	8687	7174	8593	**8883**
140	HCC	8777	6907	8285	**9091**
141	HCC	8933	7073	8493	**9272**
142	HCC	8545	6808	8129	**8887**
143	HCC	8816	7287	8779	**9035**
144	HCC	8442	6805	8130	**8775**
145	HCC	8700	6475	7905	**9013**
146	HCC	8200	6571	7845	**8487**
147	HCC	10033	9158	11021	**11130**
148	HCC	394	1682	209	**2721**
149	HCC	4614	3272	2818	**6317**
150	HCC	7215	5451	6244	**9039**

Supporting Table 3. The predicted result of 4 groups with 4 formulas of the 88-microRNA signature in the validation set.

Sample	Group	Predict value for HC	Predict value for CHB	Predict value for LC	Predict value for HCC	Accuracy (%)
1	HC	**7044**	5069	5610	6491	
2	HC	**7066**	4706	5285	6398	
3	HC	**7093**	5076	5629	6538	
4	HC	**7072**	4709	5290	6404	
5	HC	**7226**	5016	5606	6654	
6	HC	**6286**	4154	4524	5603	
7	HC	**5586**	3958	4128	4957	**100**
8	HC	**6262**	4693	5172	5805	
9	HC	**17889**	15450	18354	17390	
10	HC	**7044**	5069	5610	6491	
11	HC	**21047**	16678	20381	19461	
12	HC	**21892**	17031	20741	19931	
13	HC	**19236**	15373	18707	17828	

14	CHB	11605	**15419**	13445	12608	
15	CHB	15076	**17851**	15616	16084	
16	CHB	5069	**7044**	5610	6491	
17	CHB	4706	**7066**	5285	6398	
18	CHB	5076	**7093**	5629	6538	
19	CHB	4709	**7072**	5290	6404	
20	CHB	5016	**7226**	5606	6654	
21	CHB	13557	12197	**14499**	14023	**80**
22	CHB	15860	**16371**	16223	13315	
23	CHB	16839	**17482**	16989	13885	
24	CHB	16148	**16713**	16646	13660	
25	CHB	17134	**17769**	17215	14029	
26	CHB	13663	12150	**14461**	14049	
27	CHB	12435	10845	**12782**	12730	
28	CHB	18529	**19499**	18946	15517	
29	LC	15420	14404	**16653**	15935	
30	LC	16483	15971	**18473**	17256	
31	LC	14931	13816	**16098**	15491	
32	LC	15428	14168	**16677**	16053	
33	LC	16747	15859	**18395**	17363	
34	LC	15533	15132	**17574**	16449	
35	LC	16107	14771	**17534**	16723	
36	LC	13777	15485	**17987**	14604	
37	LC	13741	15445	**17937**	14559	
38	LC	8681	11819	**13062**	9468	
39	LC	12508	13591	**15930**	13976	
40	LC	13387	13848	**15855**	14256	
41	LC	2983	3131	**4284**	3766	
42	LC	16857	14217	**17405**	17684	**93.4**
43	LC	14805	528	**15416**	15253	
44	HCC	15688	12699	**15663**	16662	
45	HCC	17843	15853	**19076**	19571	
46	HCC	19323	16484	**20004**	20806	
47	HCC	18556	15138	**18499**	19666	
48	HCC	18612	15160	**18482**	19556	
49	HCC	18760	15803	**19196**	19919	
50	HCC	20100	16522	**20302**	21222	
51	HCC	19042	15695	**19162**	20184	
52	HCC	18647	15154	**18482**	19675	
53	HCC	19023	15259	**18746**	19791	
54	HCC	19308	15930	**19507**	20620	
55	HCC	17903	14715	**17790**	18620	

56	HCC	17166	14242	17294	**18021**
57	HCC	20114	17136	21035	**21489**
58	HCC	18941	15653	19108	**20107**
59	HCC	20871	17311	21280	**22247**
60	HCC	17506	14956	18059	**18644**
61	HCC	16130	13704	16695	**16736**
62	HCC	15218	12848	15514	**15547**
63	HCC	16059	13553	16513	**16584**

Supporting Table 4. HCC diagnostic efficiency of 88-microRNA and AFP on 213 subjects.

		**HCC case N (%)**	**Non-HCC case N (%)**	**Total cases**
**Signature prediction**	**Cancer**	80 (100)	1 (0.8)	81
	**Non-cancer**	0 (0.0)	132 (99.2)	132
**AFP prediction**	**Cancer**	51 (63.8)	21 (15.8)	72
	**Non-cancer**	29 (36.2)	112 (84.2)	141
	**Total cases**	80	133	213
